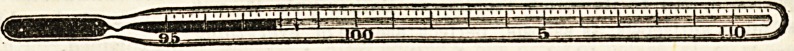# New Drugs, Appliances, and Things Medical

**Published:** 1892-01-16

**Authors:** 


### NEW DRUGS, APPLIANCES, AND THINGS MEDICAL.

[All preparations' appliances, novelties, etc., of which a notice is desired, should be sent for The Editor, to care of The Manager, 140, Strand, London, W.C.]

### NEW CLINICAL THERMOMETER: HICKS' PATENT.

Mr. Hicks, the instrument maker, of Hatton Garden, has sent us a specimen of his new patent thermometer. It has several distinctive features, which may decidedly be called improvements. The most important of these, from a practical point of view, is that the thermometer does actually indicate the exact temperature of the human body in the very brief time of half a minute, or from that to a minute and a-half at most. This is really all that ought to be demanded of a trustworthy instrument. The thermometer has been examined and reported upon by Mr. W. M. Whipple, of the Kew Observatory, who pronounces it to possess all the advantages claimed for it. The constricted neck of the old-fashioned instrument has been found by Mr. Hicks to be unnecessary, and has, therefore, been discarded. There is thus no impediment of any kind to the free upward expansion of the mercurial column. The instrument is strong, has an excellent index, and a broad, distinct indicator. It is emphatically an "improved thermometer."

## Figures and Tables

**Figure f1:**